# Photo‐induced proton release of spiropyran‐derived nanomaterials for mRNA delivery in hard‐to‐transfect cells

**DOI:** 10.1002/smo2.70042

**Published:** 2026-03-13

**Authors:** Caiwei Lu, Zhiwei Zhou, Leilei Li, Zhu Yu, Feiyu Chen, Yi Xiao, Xinfu Zhang

**Affiliations:** ^1^ State Key Laboratory of Fine Chemicals, Department of Pharmaceutical Engineering Frontiers Science Center for Smart Materials Oriented Chemical Engineering Dalian University of Technology Dalian China; ^2^ Ningbo No 2 Hospital Ningbo China; ^3^ Ningbo Institute of Dalian University of Technology Dalian University of Technology Ningbo China

**Keywords:** lipid‐like materials, messenger RNA delivery, photoacid, spiropyran

## Abstract

Messenger RNA (mRNA) therapy has been explored as a treatment for various genetic disorders. Significant progress has been made in developing mRNA delivery systems. However, efficient delivery of mRNA in hard‐to‐transfect cells remains a considerable challenge. Here, we constructed a series of spiropyran‐derived lipid‐like materials, named SPALs, to enhance mRNA delivery efficiency in cells that are difficult to transfect. In this design, we focused on strengthening the internal escape mechanism of mRNA nanoparticles via photo‐induced proton production by SPALs. Specifically, the spiropyran structure can undergo ring‐closing isomerization under light irradiation (460 nm) and produce a proton. The protons then mediate the protonation of tertiary amines in the lipid tail in situ, which subsequently disrupts the potential and size of nanoparticles and finally promotes their endosome escape. It ultimately enhances mRNA expression in live cells. SPALs are demonstrated to reduce the pH value up to 0.53 pH units after light irradiation in solution. They are also proven to decrease the pH value after light irradiation as observed through fluorescence ratio imaging in live cells. After screening the materials, we identified SPAL1 as a lead material for increasing the enhanced green fluorescent protein‐positive rate in Raw 264.7 cells by 22% after light irradiation, from 59% to 72%. This transfection efficiency is significantly higher than that of the lipo3000 in the same cell line. Consequently, we developed a new strategy to improve mRNA transfection efficiency, which merits further evaluation and development for mRNA‐based therapy.

## Graphical abstract

Light‐induced proton release from spiropyran can disrupt the stability of both nanoparticles and endosomal membranes, thereby promoting endosomal escape and ultimately enhancing transfection performance.

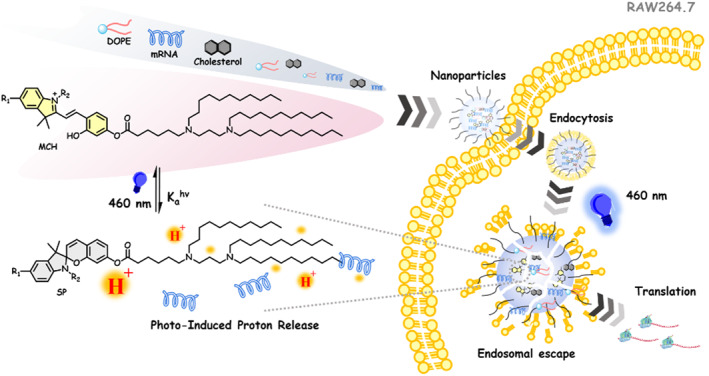

## 1 INTRODUCTION

Messenger RNA (mRNA) therapy has emerged as a promising and transformative treatment for various diseases by directly regulating protein expression.[^1^, ^2^, ^3^, ^4^, ^5^, ^6^] Therapeutic proteins can be supplemented using in vitro transcribed mRNA through the intrinsic protein synthetic pathway.^[7]^ However, mRNA is difficult to be directly taken up and utilized by living cells due to its inherent instability against ribonuclease enzymes and strong polyanionic nature. Therefore, the development of effective mRNA delivery systems is crucial for protecting mRNA from degradation and enhancing its cellular uptake. Among the reported platforms, lipid‐like materials, characterized by a core structure containing multiple tertiary amines and hydrophobic long‐chain alkyl side groups, have emerged as a prominent class of mRNA delivery vectors. These materials can efficiently encapsulate mRNA via electrostatic and hydrophobic interactions, forming lipophilic nanoparticles that facilitate mRNA delivery into living cells.[^8^, ^9^, ^10^] The promising delivery efficiency encourages us to explore new lipid‐like nanoparticles (LLNs) and their therapeutic potential further.

However, as with most reported mRNA delivery platforms, LLNs exhibit varying mRNA expression efficiency across different cell lines. The intracellular working mechanisms of these lipid‐like compounds remain poorly understood. Enhancing the cellular delivery efficiency of lipid‐like materials, particularly in cell lines with inherently low transfection or expression efficiency, remains a significant challenge. According to the literature, endosomal escape is a critical prerequisite for efficient mRNA expression.^[11]^ To address the barrier to endosomal escape, various strategies have been explored. These include the incorporation of functional peptides or proteins that disrupt endosomal membranes,^[12]^ the application of mechanical stress^[13]^ or osmotic pressure,^[14]^ ultrasound‐induced cavitation,^[15]^ and the use of reactive oxygen species^[16]^ (e.g., singlet oxygen) to compromise endosomal integrity. Generally, breaking endosomal integrity is beneficial for enhancing mRNA expression.

We aim to enhance mRNA delivery efficiency by focusing on the structural properties of lipid‐like materials. Their core tertiary amines are ionizable, providing the primary electrostatic force for binding to mRNA. According to the workflow for fabricating LLNs, moderate protonation of these tertiary amines may favor their binding with mRNA and the stability of the LLNs. On the contrary, over‐protonation of these tertiary amines may disrupt the structural integrity, size, and surface charge of the nanoparticles, thereby facilitating endosomal escape and enhancing mRNA delivery efficiency.^[17]^ Herein, we propose incorporating spiropyran moieties into lipid‐like molecules enabling light‐induced acid production and consequently promoting endosomal escape through in situ protonation. We designed and synthesized six spiropyran‐lipid derivatives with varying substituents and confirmed their ability to generate acid upon light irradiation by measuring the pH change in solution and using ratiometric imaging. We also screened protein expression using an in vitro assay and identified a lead material, spiropyran‐derived lipid‐like material (SPAL1), that shows the highest enhancement.

## 2 RESULTS AND DISCUSSION

### 2.1 Design and synthesis of SP‐Lipids

**FIGURE 1:**
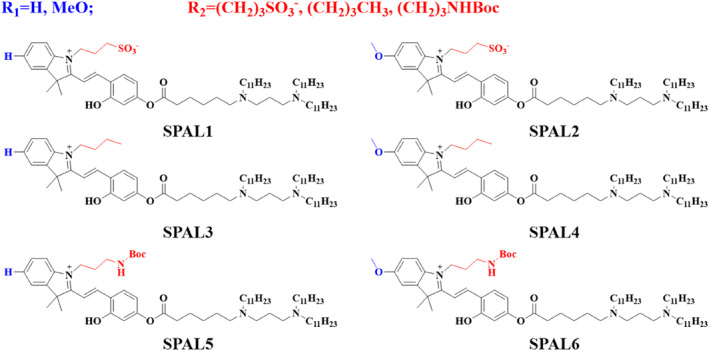
Molecular structures of the designed spiropyran‐lipid.

**FIGURE 2:**
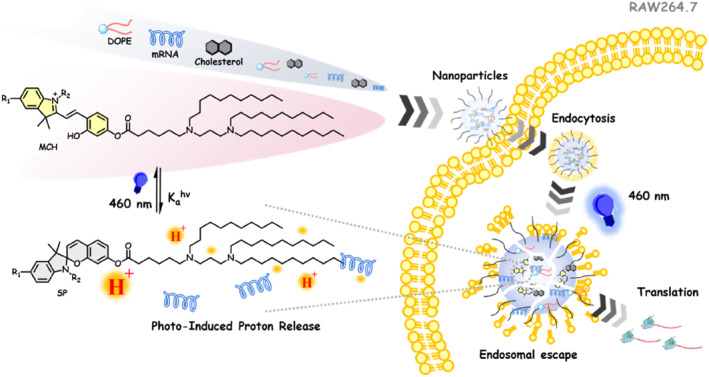
Schematic illustration of the working mechanism of the target compound.

We first designed and synthesized a small library of SPAL1‐6 (Figure 1) by connecting a spiropyran scaffold and a lipid‐like tail via ester linkages. The spiropyrans will function as photoacid generators undergoing reversible structural isomerization upon exposure to light.[^18^, ^19^, ^20^, ^21^] Specifically, spiropyran derivatives can switch from an open form to a closed one while generating a proton upon irradiation with 460 nm light. We modified substituents at two positions of the spiropyran (Figure 2, R_1_ and R_2_) to evaluate their effects on proton dissociation behavior. In a weakly acidic environment, the isomerization rate of spiropyran increases. We selected H or OMe (an electron‐donating group) at the R_1_ position and a butyl, amide, or sulfonic acid group at the R_2_ position. We synthesized 6 compounds for comparison (Figures S11–S37, nuclear magnetic hydrogen spectroscopy and high‐resolution mass spectrometry). By modifying the R_1_ substituent, the spiropyran's response range can be tuned. Altering the R_2_ substituent adjusts the overall electronic properties of the spiropyran moiety, thereby modulating the cellular uptake and transfection efficiency of the nanoparticles. The series of molecules was synthesized via a modular approach with high yields, and all compounds were fully characterized.

### 2.2 Photo‐induced isomerization and proton generation of the spiropyran cores

**FIGURE 3:**
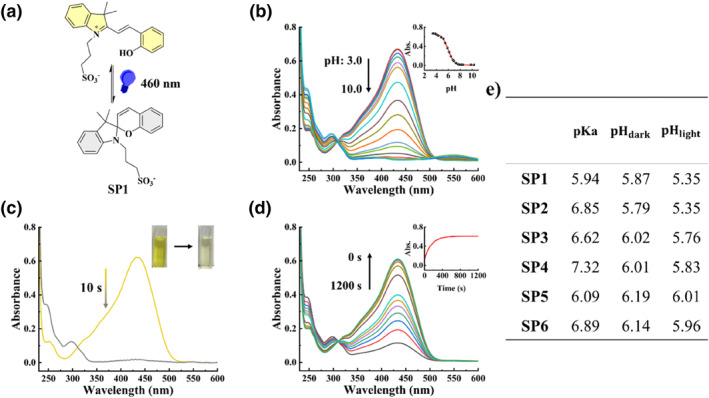
Absorption spectrum of spiropyran. (a) SP1 ring‐opening and ring‐closing schematic diagram. (b) Absorption spectra under different pH conditions (yellow, *λ*_MCH_ = 430–460 nm; purple, *λ*_MC_ = 500–600 nm). Experimental conditions: (SP) = 30 μM, pH 3–10, *T* = 25°C. (c) Absorption spectra before and after illumination, *hv* (blue, 460 nm, 5 W, 10 s). (d) The changes of absorption spectra after illumination, time: 1200 s. (e) Related parameters of the solution of SPs before and after illumination.

We initially investigated the pH response of these spiropyrans and the photo‐induced isomerization and proton generation effect of the spiropyran cores to demonstrate their potential to protonize tertiary amines in the lipid tail. According to a previous study, the photoswitch from ring‐opening spiropyran to ring‐closing spiropyran involves *cis*–*trans* isomerization and ring‐closing steps. The second step is also affected by environmental pH. As shown in Figure 1, the photoinduced isomerization of spiropyrans occurs between the ring‐opening and ring‐closing states, which possess different π‐conjugates, corresponding to distinct absorption spectrum features (wavelengths). We therefore performed pH titrations of these spiropyrans in an ethanol/water mixture and recorded the corresponding UV–Vis absorption spectra to determine their working pH range. As the pH decreased, the absorption peak around 425 nm increased significantly, while that around 280 nm decreased. Based on the titration curves, we calculated the pKa value (Figure 3e) of the photo‐induced ring closing process. We observed that the R_1_ substituent on the aromatic ring significantly influences the pKa. For instance, the introduction of an electron‐donating methoxy group at the R_1_ position resulted in a lower pKa value of SP1 than SP2 (Figure 3e). From the perspective of pKa, SP1 and SP5 have an ideal pKa of ∼6.0, which is desirable as it would enable applications near endosomal pH values. An increase in pH increases the rate of isomerization of spiropyran.^[22]^

We then investigated the photoinduced isomerization and proton‐generating effects of these spiropyran cores. We irradiated the spiropyran scaffolds in an ethanol/water mixture with 460 nm light and recorded their UV–Vis absorption spectra over time. As shown in Figure 3 and Figures S1–S5, the absorption peak around 460 nm disappeared within an average of 5–10 s upon light irradiation, indicating that the photo‐induced ring closing transformation takes place efficiently and rapidly. Upon light irradiation, the solution exhibited evident fading, turning from yellow to nearly transparent. Its rapid response enables the rapid release of protons, thereby altering the acidity of the endosomal microenvironment and facilitating endosomal disruption and the subsequent release of nucleic acids. Furthermore, we measured the pH of the spiropyran solution before and after irradiation and observed a decrease of 0.18–0.52 units, indicating the generation of protons upon irradiation (Figure 3e). Among these spiropyrans, SPAL1 showed the most significant pH decrease of 0.52. These features align with our expectations for using photoinduced protons to tune the LLN structure. Additionally, the absorption peak around 460 nm gradually recovered to its original level within 20 min, indicating that the ring‐closing spiropyran can revert to the ring‐opening form spontaneously in the dark.

### 2.3 Characterization of SPAL LLNS with or without light irradiation

**FIGURE 4:**
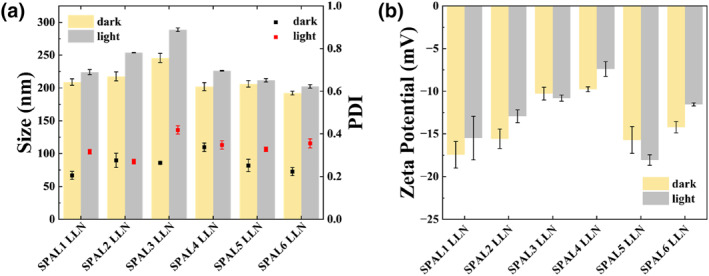
The particle size and zeta potential of SP LLNs. (a) Changes in nanoparticle size and polydispersity index before and after light irradiation (NPs solution/H_2_O = 1/50, v/v). (b) Changes in zeta potential before and after light irradiation (NPs solution/H_2_O = 1/50, v/v). LLNs, lipid‐like nanoparticles; NPs, nanoparticles.

Then, we investigated the characteristic changes in SPAL LLNs before and after light irradiation, including particle size, polydispersity index (PDI), and zeta potential. We formulated SPALs into SPAL LLN formulations according to previously reported methods.^[9]^ Formulation components include SPALs, 1,2‐dioleoyl‐sn‐glycero‐3‐phosphoethanolamine (DOPE), cholesterol, 1,2‐dimyristoyl‐racglycero‐3‐methoxypolyethylene glycol‐2000 (DMG‐PEG2000), and enhanced green fluorescent protein (eGFP) mRNA. The molar ratio of components within SPAL LLN formulations was 20/30/40/0.75 (SPALs/DOPE/cholesterol/DMG‐PEG2000). The formula with this ratio can successfully encapsulate mRNA (Figure S6). To prepare these formulations, mRNA was dissolved in citrate buffer, while other components were dissolved in the ethanol phase. Through rapid mixing of the above two phases by mechanical pipetting, SPAL LLN formulations were afforded. The sizes of SPAL1‐6 LLNs were measured to be 200–300 nm with a PDI lower than 0.4 before light irradiation (Figure 4a). The zeta potentials range from −20 to −5 mV (Figure 4b). Upon exposure to light (460 nm, 300 s), the sizes of SPAL1‐6 LLNs increase slightly, accompanied by noticeable changes in zeta potentials. The increase in nanoparticle size accompanied by changes in surface potential suggests that the interactions between the internal lipid‐like materials and the encapsulated mRNA may have been altered, indicating a partial loss of structural integrity and reduced particle stability. Consistently, the increase in the PDI further supports a transition of the nanoparticle population from a relatively homogeneous state to a more heterogeneous one. We propose that the protons generated upon light irradiation decrease the local microenvironmental pH within the nanoparticles leading to structural perturbations of the particle assembly. The pH‐induced destabilization observed at the nanoparticle level likely reflects partial nanoparticle disassembly accompanied by enhanced endosomal disruption and escape, ultimately contributing to increased transfection efficiency.

### 2.4 Screening of SPALs for mRNA delivery with or without light irradiation

**FIGURE 5:**
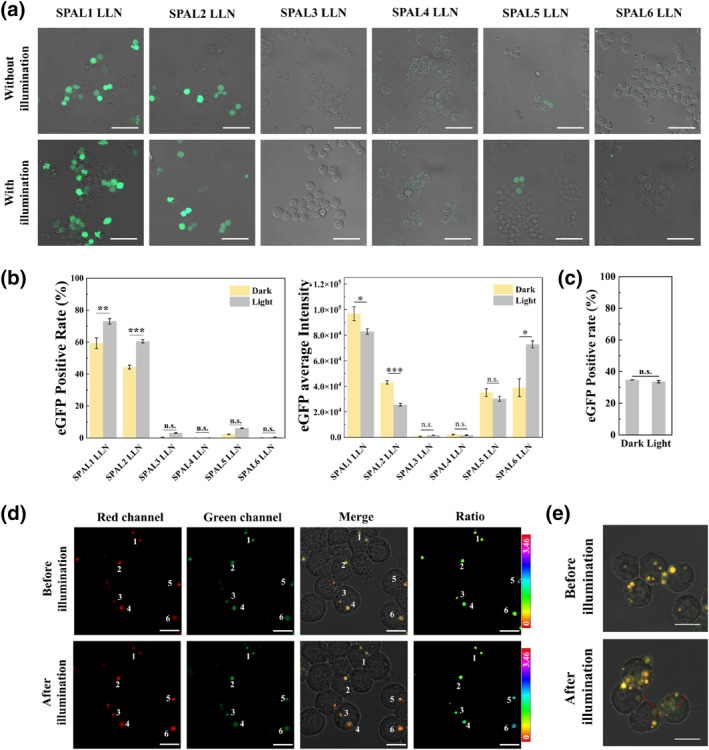
Confocal microscopy and flow cytometry analysis. (a) Transfection of eGFP mRNA using spiropyran‐lipid‐like nanoparticles under light irradiation (460 nm, 5 min, 5 W); *λ*_ex_ = 488 nm, *λ*_em_ = 509 nm, bar = 60 μm. (b) The positive rate and mean fluorescence intensity of green fluorescent protein expression in flow cytometry. (*p < 0.05, **p < 0.01, ***p < 0.001.) (c) Transfection of Lipo3000 before and after illumination. (d) Fluorescence ratiometric (red/green) images of cells treated with nanoparticles composed of 4% ratiometric probe‐lipid materials encapsulating CtDNA. *hv* (blue, 460 nm, 5 W, 5 min), *λ*_ex_ = 488 nm, green channel: *λ*_em_ = 500–521 nm, red channel: *λ*_em_ = 50–620 nm, bar = 20 μm. (e) Fluorescence images (red and green merged) of cells treated with nanoparticles composed of 4% ratiometric probe‐lipid materials encapsulating CtDNA *hv* (blue, 460 nm, 5 W, 5 min), *λ*_ex_ = 488 nm, green channel: *λ*_em_ = 500–521 nm, red channel: *λ*_em_ = 50–620 nm, bar = 20 μm. eGFP, enhanced green fluorescent protein.

Then, we assessed the mRNA‐delivery capability of SPAL LLNs encapsulating eGFP mRNA in RAW 264.7 cells. This cell line is a murine monocytic macrophage leukemia cell. Its primary role is the identification, phagocytosis, and destruction of foreign pathogens (viruses and bacteria) and other substances. This highly active phagocytic nature results in the constant internalization and lysosomal degradation of extracellular material. Thus, it is inherently difficult for foreign liposomes to achieve successful endosomal escape in these cells. This intrinsic feature results in a low transfection rate (<30%) even when using commercial mRNA delivery reagents such as lipofectamine 3000. In this assay, we treated cells with freshly prepared LLNs at a dose of 125 ng per well in a 96‐well plate for 9 h. For the irradiation group, cells were irradiated for 5 min with 460 nm LED light at the third hour of transfection. All cells were imaged by confocal microscopy to compare transfection efficiency based on eGFP green fluorescence. As shown in Figure 5a, we observed intense green fluorescence in the SPAL1 LLN and SPAL2 LLN groups with and without irradiation. We found an apparent increase in GFP fluorescence for the light irradiation group for both SPAL1 and SPAL2 LLNs. This result suggests that the photo‐induced ring‐closing isomerization of spiropyran increases protein expression. We further propose that the protons generated upon light activation may associate with sulfonate groups to form sulfonic acids whose greater acidity may facilitate more efficient endosomal escape. As can be seen from Figure 3e, the pH change of the sulfonic acid chain molecules in solution after light irradiation is approximately 0.3–0.5, which is higher than that of the other two substituted chains. This also supports that increased acidity facilitates escape. We can demonstrate using an methyl thiazolyl tetrazolium assay that there is no significant difference in cell toxicity due to light (Figure S7).

We subsequently conducted flow cytometry to quantitatively assess the mRNA delivery capability. We treated cells with freshly prepared LLNs at a dose of 375 ng per well in a 48‐well plate for 9 h. For the irradiation group, cells were irradiated for 5 min with 460 nm LED light at the third hour of transfection. Cells were collected and analyzed using flow cytometry. As shown in Figure 5b and Figure S8, SPAL1 and SPAL2 LLNs exhibited the highest rates of eGFP‐positive results, at 59% and 44%, respectively. In the light‐irradiation groups, the rates of eGFP‐positive results in SPAL1 and SPAL2 LLNs increased to 72% and 61%, respectively (Table S1). This result reveals that the SPAL1 and SPAL2 LLNs exhibited an enhanced positive rate of eGFP of 22% and 39% after light irradiation, respectively. We performed flow cytometry analysis using the commercial transfection reagent Lipo3000 at equal RNA concentrations, and the eGFP‐positive rate was 34%. The rates of eGFP‐positive results of SPAL1 and SPAL2 were significantly higher than those achieved with Lipo3000. We also examined the fluorescence intensity of transfected positive cells. The average fluorescence intensity of eGFP decreased slightly in the light‐irradiation groups compared with the non‐irradiation groups. To evaluate light‐induced biological or physicochemical effects. We performed flow cytometry on cells transfected with the commercial reagent Lipo3000 before and after light irradiation. As shown in Figure 5c and Figure S9, no significant difference in transfection rate was observed before or after light exposure. These results indicate that light‐induced photon release promotes the disruption of particles and endosomes, thereby boosting mRNA transfection rates.

Given the above result, we sought to directly demonstrate proton generation via light irradiation in live cells. We formulated SPAL1 LLNs by adding 4% (weight% vs. SPAL1) of pH‐sensitive lipid‐like materials RPHL (Figure S10). RPHL is a ratiometric pH probe linked to a lipid‐like tail. It exhibited green fluorescence from BODIPY predominantly at neutral and alkaline pH, while red emission was predominantly observed at acidic pH. The ratio of red fluorescence (I_red_) to green fluorescence (I_green_) can be used to indicate pH semi‐quantitatively. We treated cells with freshly prepared LLNs in a 96‐well plate for 3 h. Cells were imaged by confocal microscopy immediately after light irradiation. Quantitative analysis of fluorescence in cells showed that the average fluorescence ratio (I_red_/I_green_) in endosomes increased from 1.30 to 1.70 after light irradiation (Figure 5d and Table S2). This increase in the ratio indicates a decrease in endosomal pH, suggesting that light‐irradiation‐induced ring‐closing isomerization of spiropyran generates protons that lower endosomal pH. At the same time, we found that the fluorescence of the inner body after illumination gradually changed from clear dots to blurred edges (Figure 5e), indicating that the particle structure had changed and accelerated endosome escape.

## 3 CONCLUSION

In conclusion, we synthesized and screened a series of spiropyran‐derived lipid‐like nanomaterials. We demonstrated photo‐induced isomerization, proton generation, and delivery efficiency, and compared them with those of lipo3000. We showed that SPAL1‐6 undergoes a ring‐closing reaction upon illumination at 460 nm, generating protons and decreasing the local microenvironmental pH. This process facilitates enhanced endosomal escape. We constructed nanoparticles via self‐assembly of the delivery material with GFP‐encoding mRNA and transfected them into macrophage cells. We observed that light irradiation increased the transfection efficiency of SPAL1 LLNs from 59% to 72%. This number is significantly higher than that of the lipo3000 (34%) in the same cell line. These results demonstrate that light‐induced proton release from spiropyran can destabilize both nanoparticles and endosomal membranes, thereby promoting endosomal escape and ultimately enhancing transfection performance. Therefore, our design of new lipid‐like nanomaterials further improved delivery efficiency in RAW 264.7 cells. Overall, the lead SAPL1 LLNs merit further investigation and application.

## 4 EXPERIMENTAL SECTION/METHODS

All chemicals were obtained from commercial suppliers and used without further purification. Melting points were determined using an uncorrected melting point apparatus. ^1^H NMR and ^13^C NMR were measured in CDCl_3_ with tetramethylsilane as internal reference. Coupling constants (J) are given in Hz. Column chromatography was performed with silica gel (200–300 mesh). All solvent mixtures are given as volume/volume ratios. Fluorescence quantum yields were determined using fluorescein as a reference.

RAW 264.7 (Macrophage cells) were cultured in dulbecco's modified eagle medium (DMEM) supplemented with 10% fetal bovine serum in an atmosphere of 5% CO_2_ and 95% air at 37°C. For mRNA transfection studies, macrophage cells in the exponential growth phase were plated into a 35‐mm cross‐shaped compartmentalized confocal dish containing 500 μL DMEM per compartment. The media was removed after incubation at 37°C with 5% CO_2_ for 24 h. A freshly prepared nanoparticle stock solution was then added to the DMEM culture medium. After incubation for 3.5 h, the cells were exposed to 460 nm LED light for 5 min, followed by an additional 6‐h incubation. Confocal imaging was then performed using a confocal laser scanning microscope.

In terms of cell culture procedures, the experimental procedures for flow cytometry were essentially the same as those for confocal microscopy. The culture medium was removed after washing with 500 mL of phosphate buffer saline, and the cells were resuspended in 300 μL of culture medium in a centrifuge tube. Samples were illuminated with a sapphire laser at 488 nm on a FACScan flow cytometer. Each group of samples detected 10,000 cells in 1 min. Analysis of flow cytometry data with FlowJo software.

## CONFLICT OF INTEREST STATEMENT

The authors declare no conflicts of interest.

## ETHICS STATEMENT

No animal or human experiments were involved in this study.

## Supporting information

Supporting Information S1

## Data Availability

The data that support the findings of this study are available on request from the corresponding author. The data are not publicly available due to privacy or ethical restrictions.

## References

[1] S. Qin , X. Tang , Y. Chen , K. Chen , N. Fan , W. Xiao , Q. Zheng , G. Li , Y. Teng , M. Wu , X. Song , Signal Transduction Targeted Ther. 2022, 7, 166. 10.1038/s41392-022-01007-w PMC9123296 35597779

[2] N. Pardi , M. J. Hogan , F. W. Porter , D. Weissman , Nat. Rev. Drug Discovery 2018, 17, 261. 29326426 10.1038/nrd.2017.243 PMC5906799

[3] K. Itaka , Nihon Yakurigaku Zasshi 2016, 148, 190. 27725567 10.1254/fpj.148.190

[4] J. C. Kaczmarek , A. K. Patel , K. J. Kauffman , O. S. Fenton , M. J. Webber , M. W. Heartlein , F. DeRosa , D. G. Anderson , Angew Chem., Int. Ed. Engl. 2016, 55, 13808. 27690187 10.1002/anie.201608450 PMC5279893

[5] K. Ramani , Q. Hassan , B. Venkaiah , S. E. Hasnain , D. P. Sarkar , Proc. Natl. Acad. Sci. U. S. A. 1998, 95, 11886. 9751760 10.1073/pnas.95.20.11886 PMC21735

[6] X. Tang , H. Peng , P. Xu , L. Zhang , R. Fu , H. Tu , X. Guo , K. Huang , J. Lu , H. Chen , Z. Dong , L. Dai , J. Luo , Q. Chen , Mol. Ther. Oncolytics 2022, 24, 707. 35317516 10.1016/j.omto.2022.01.013 PMC8913249

[7] M. S. Khan , S. A. Baskoy , C. Yang , J. Hong , J. Chae , H. Ha , S. Lee , M. Tanaka , Y. Choi , J. Choi , Nanoscale Adv. 2023, 5, 1853. 36998671 10.1039/d2na00795a PMC10044484

[8] Z. Chen , Y. Tian , J. Yang , F. Wu , S. Liu , W. Cao , W. Xu , T. Hu , D. J. Siegwart , H. Xiong , J. Am. Chem. Soc. 2023, 145, 24302. 37853662 10.1021/jacs.3c09143

[9] X. Zhang , W. Zhao , G. N. Nguyen , C. Zhang , C. Zeng , J. Yan , S. Du , X. Hou , W. Li , J. Jiang , B. Deng , D. W. McComb , R. Dorkin , A. Shah , L. Barrera , F. Gregoire , M. Singh , D. Chen , D. E. Sabatino , Y. Dong , Sci. Adv. 2020, 6. 10.1126/sciadv.abc2315 PMC7442477 32937374

[10] B. Li , X. Luo , B. Deng , J. Wang , D. W. McComb , Y. Shi , K. M. Gaensler , X. Tan , A. L. Dunn , B. A. Kerlin , Y. Dong , Nano Lett. 2015, 15, 8099. 26529392 10.1021/acs.nanolett.5b03528 PMC4869688

[11] S. A. Smith , L. I. Selby , A. P. R. Johnston , G. K. Such , Bioconjugate Chem. 2019, 30, 263. 10.1021/acs.bioconjchem.8b00732 30452233

[12] R. Mout , M. Ray , T. Tay , K. Sasaki , G. Yesilbag Tonga , V. M. Rotello , ACS Nano 2017, 11, 6416. 28614657 10.1021/acsnano.7b02884 PMC5766003

[13] A. Tamura , M. Oishi , Y. Nagasaki , Biomacromolecules 2009, 10, 1818. 19505137 10.1021/bm900252d

[14] M. Massignani , I. Canton , T. Sun , V. Hearnden , S. Macneil , A. Blanazs , S. P. Armes , A. Lewis , G. Battaglia , PLoS One 2010, 5, e10459. 20454666 10.1371/journal.pone.0010459 PMC2862714

[15] M. Chen , J. Cen , Q. Shi , B. Shao , J. Tan , X. Ye , Z. He , Y. Liu , G. Zhang , J. Hu , J. Bao , S. Liu , Angew Chem., Int. Ed. Engl. 2025, 64, e202500878. 39878170 10.1002/anie.202500878

[16] T. Ohtsuki , S. Miki , S. Kobayashi , T. Haraguchi , E. Nakata , K. Hirakawa , K. Sumita , K. Watanabe , S. Okazaki , Sci. Rep. 2015, 5, 18577. 26686907 10.1038/srep18577 PMC4685267

[17] A. Akinc , A. Zumbuehl , M. Goldberg , E. S. Leshchiner , V. Busini , N. Hossain , S. A. Bacallado , D. N. Nguyen , J. Fuller , R. Alvarez , A. Borodovsky , T. Borland , R. Constien , A. de Fougerolles , J. R. Dorkin , K. Narayanannair Jayaprakash , M. Jayaraman , M. John , V. Koteliansky , M. Manoharan , L. Nechev , J. Qin , T. Racie , D. Raitcheva , K. G. Rajeev , D. W. Sah , J. Soutschek , I. Toudjarska , H. P. Vornlocher , T. S. Zimmermann , R. Langer , D. G. Anderson , Nat. Biotechnol. 2008, 26, 561. 18438401 10.1038/nbt1402 PMC3014085

[18] L. Kortekaas , W. R. Browne , Chem. Soc. Rev. 2019, 48, 3406. 31150035 10.1039/c9cs00203k

[19] T. Khalil , A. Alharbi , C. Baum , Y. Liao , Macromol. Rapid Commun. 2018, 39, e1800319. 29924433 10.1002/marc.201800319

[20] P. K. Patel , V. K. Johns , D. M. Mills , J. E. Boone , P. Calvo‐Marzal , K. Y. Chumbimuni‐Torres , Electroanalysis 2015, 27, 677.

[21] L. Wimberger , S. K. K. Prasad , M. D. Peeks , J. Andreasson , T. W. Schmidt , J. E. Beves , J. Am. Chem. Soc. 2021, 143, 20758. 34846132 10.1021/jacs.1c08810

[22] J. Liu , W. Tang , L. Sheng , Z. Du , T. Zhang , X. Su , S. X. Zhang , Chem. Asian J. 2019, 14, 438. 30536732 10.1002/asia.201801687

