# Supplementary material for: Photo‐induced proton release of spiropyran‐derived nanomaterials for mRNA delivery in hard‐to‐transfect cells
Source: Smart Mol. 2026 Mar 13;4(2):e70042. doi: 10.1002/smo2.70042 (PMC13317551; doi:10.1002/smo2.70042)
Supplement: Supplementary file 1 — Supporting Information S1 [file SMO2-4-e70042-s001.docx]

**Photo-Induced Proton Release of Spiropyran Derived Nanomaterials for mRNA Delivery in Hard-to-Transfect Cells**

Caiwei Lu ^1^, Zhiwei Zhou^1^, Leilei Li^1^, Zhu Yu^1^, Feiyu Chen ^2,^*, Yi Xiao ^1,^* and Xinfu Zhang ^1,2,^*

^1^ State Key Laboratory of Fine Chemicals, Frontiers Science Center for Smart Materials Oriented Chemical Engineering, Department of Pharmaceutical Engineering, Dalian University of Technology, Dalian 116024, China; ^2^ Ningbo No2 Hospital, No.41 Xibei Street, Ningbo 315000, China; ^3^ Ningbo Institute of Dalian University of Technology, Dalian University of Technology, Ningbo 315000, China

E-mail: cfy979619@sina.com; xiaoyi@dlut.edu.cn; zhangxinfu@dlut.edu.cn

**Supplemental Experimental Procedures**

1. **General methods and materials**

All chemicals were obtained from commercial suppliers and used without further purification. ^1^H NMR and ^13^C NMR spectra were measured in CDCl_3_ and DMSO-d6, using TMS as the internal reference. Coupling constants (J) are given in Hz. Column chromatography was performed with silica gel (200-300 mesh). Fluorescence quantum yields were determined using fluorescein as a reference.

1. **The synthesis procedure of SPAL**

Synthetic route:

Synthesis of **compound 2**:

A solution of **compound 1** (30 g, 404.81 mmol) in 300 mL CH₂Cl₂ was added (Boc)₂O (15 g, 68.73 mmol) in 150 mL CH₂Cl₂ using a constant pressure drip funnel. The reaction was stirred at room temperature for 24 hours. Upon completion, the organic phase was dried over Na_2_SO_4_ after being washed twice with a saturated sodium bicarbonate solution and twice with brine, and then concentrated under reduced pressure. The product was used for the next step without purification.

Synthesis of **compound 3**:

A solution of **compound 2** (1.5 g, 8.96 mmol), ethyl 6-bromohexanoate (1 g, 4.48 mmol), and 1.5 ml Et_3_N in 5 ml acetonitrile was added under 0 °C. The mixture was warmed to room temperature and stirred for 48 hours. Upon completion, the organic phase was dried over Na_2_SO_4_ after being washed with brine twice and then concentrated under reduced pressure. The residue was purified by column chromatography (DCM/Ultra = 100/0- 90/10, Ultra: DCM/MeOH/NH_3_∙H_2_O = 75:22:3) to afford **compound 3** (665 mg, 47%).

Synthesis of **compound 4**:

A solution of **compound 3** (0.5 g, 1.58 mmol) in 1 mL of CH_2_Cl_2_ was added to 2 mL of trifluoroacetic acid. The reaction was stirred for 30 min at room temperature. After TLC analysis revealed the absence of compound 3, the mixture was concentrated under reduced pressure. The solution pH was adjusted to alkaline conditions with triethylamine. The reaction was initiated by adding undecanal (1.35 g, 7.90 mmol) and STAB (1.67 g, 7.90 mmol) to 10 mL of THF. The mixture was stirred for 10 hours at room temperature. Upon completion, the organic phase was dried over Na_2_SO_4_ after being washed with brine twice and then concentrated under reduced pressure. The residue was purified by column chromatography (DCM/ Ultra = 100/0- 88/12, Ultra: DCM/MeOH/NH3∙H2O = 75:22:3) to afford **compound 4** (534 mg, 50%). ^1^H NMR (400 MHz, CDCl3) δ 4.11 (q, *J* = 7.1 Hz, 2H), 2.81 (s, 2H), 2.76 – 2.69 (m, 4H), 2.67 (s, 1H), 2.58 (s, 4H), 2.29 (t, *J* = 7.4 Hz, 2H), 1.92 (s, 2H), 1.63 (dt, *J* = 14.7, 7.3 Hz, 6H), 1.57- 1.45 (m, 4H), 1.24 (s, 54H), 0.87 (t, *J* = 6.7 Hz, 9H).

Synthesis of **compound 5**:

A solution of **compound 4** (500 mg, 0.73 mmol) in 3 mL MeOH and 3 mL THF was treated with 2 mL of 1.5 M NaOH. The reaction was stirred at 80 °C for 4 hours. Upon completion, the product was extracted into 150 ml CH_2_Cl_2_. The organic phase was dried over Na_2_SO_4_ and used for the next step without purification.

Synthesis of **compound 7a**

A solution of 2,3,3-trimethyl-3H-indole (1 g, 6.28 mmol) and 1,2-oxathiolane 2,2-dioxide (0.9 g, 7.37 mmol) was added to 10 mL of toluene. The reaction was stirred at 120 °C for 12 hours. Upon completion, the product was extracted into cool acetone. A pale purple solid precipitated from the reaction mixture and was collected by filtration. The pale purple solid is **compound 7a** (0.81 g, 46%). **Compound 7d** was synthesized using an analogous method to that of **compound 7a**.

Synthesis of **compound 7b**

A solution of 2,3,3-trimethyl-3H-indole (1 g, 6.28 mmol) and 1-iodobutane (2.5 ml, 21.97 mmol) was added to 10 mL of acetonitrile. The reaction was stirred at 85 °C for 12 hours. Upon completion, the organic phase was concentrated under reduced pressure. The residue was purified by column chromatography (DCM/MeOH = 100/0-40/1) to afford **compound 7b** (320 mg, 24%). **Compound 7e** was synthesized using an analogous method to that of **compound 7b.**

Synthesis of **compound 7c**

A solution of 2,3,3-trimethyl-3H-indole (1 g, 6.28 mmol) and 3-bromopropan-1-amine hydrobromide (1.37 g, 6.28 mml) was added in 10 ml acetonitrile. The reaction was stirred at 85 °C for 12 hours. Upon completion, the organic phase was concentrated under reduced pressure. A pale purple solid precipitated from the reaction mixture and was collected by filtration. A solution of the pale purple solid in 5 mL CHCl3 was added, followed by (Boc)_2_O (1.99 g, 9.74 mmol) and DIPEA (1.18 g, 9.74 mmol). The reaction was stirred at 68 °C for 2 hours. The organic phase was concentrated under reduced pressure after washing with diethyl ether. The reaction mixture was concentrated under reduced pressure to afford **compound 7c** (705 mg, 49%) as a purplish-red oil. **Compound 7c** was synthesized using an analogous method to that of **compound 7f**.

Synthesis of **compound 8a**

A solution of **compound 7a** (200 mg, 0.71 mmol) and 2,4-dihydroxybenzaldehyde (98.18 mg, 0.71 mml) was added in 5 ml EtOH. The reaction was stirred at 80 °C for 12 hours. Upon completion, the organic phase was concentrated under reduced pressure. The orange-red solid is **compound 7a** (159 mg, 56%). **Compound 8a-8f** was synthesized using a similar method to that of **compound 8a**.

**Compound 8a:**  ^1^H NMR (500 MHz, DMSO-*d*_6_) δ 11.13 (s, 1H), 10.91 (s, 1H), 8.53 (d, *J* = 16.0 Hz, 1H), 8.18 (d, *J* = 9.5 Hz, 1H), 7.89 (d, *J* = 8.0 Hz, 1H), 7.79 (d, *J* = 7.4 Hz, 1H), 7.68 – 7.42 (m, 3H), 6.53 – 6.38 (m, 2H), 4.68 (t, *J* = 8.0 Hz, 2H), 2.13 (t, *J* = 7.6 Hz, 2H), 1.73 (s, 6H). HRMS (ES, *m/z*): [M+H]^+^ calcd for C_21_H_24_NO_5_S^+^,402.1299; found, 402.1372. [M+Na]^+^ calcd. for C_21_H_23_NO_5_SNa^+^, 424.1189; found, 424.1195.

Synthesis of **compound SPAL**

A solution of compound 8a (30 mg, 0.75 mmol), compound 5 (50 mg, 0.77 mmol), EDC (22 mg, 1.4 mmol), and DMAP (9 mg, 0.73 mmol) was added in 1 ml CH2Cl2. The mixture was stirred at room temperature for 24 hours. Upon completion, the organic phase was concentrated under reduced pressure. The residue was purified by column chromatography (DCM/Ultra = 100/0-3/1, Ultra: DCM/MeOH/NH**_3_**∙H**_2_**O = 75:22:3) to afford **compound SPAL1** (51 mg, 66%). Compound 8a-8f was synthesized using an analogous method to that of **compound SPAL1-6.**

**SPAL1:** ^1^H NMR (500 MHz, Chloroform-*d*) δ 7.13 – 7.06 (m, 1H), 7.01 (dd, *J* = 7.9, 6.1 Hz, 2H), 6.81 – 6.73 (m, 2H), 6.56 (dd, *J* = 8.0, 2.3 Hz, 2H), 6.47 (d, *J* = 2.2 Hz, 1H), 5.60 (d, *J* = 10.2 Hz, 1H), 2.96 (d, *J* = 55.5 Hz, 8H), 2.79 – 2.75 (m, 2H), 2.51 (td, *J* = 6.4, 2.9 Hz, 2H), 2.19 – 2.07 (m, 2H), 1.76 (t, *J* = 7.1 Hz, 3H), 1.47 – 1.42 (m, 2H), 1.37 – 1.18 (m, 66H), 1.14 (s, 3H), 0.88 (d, *J* = 7.1 Hz, 9H). HRMS (ESI, *m/z*): [M+H]^+^ calcd for C_63_H_108_N_3_O_6_S^+^,1034.7970; found, 1034.7968.

**SPAL2**: ^1^H NMR (400 MHz, Chloroform-*d*) δ 7.08 (t, *J* = 7.7 Hz, 1H), 6.99 (dd, *J* = 13.3, 7.7 Hz, 2H), 6.77 (t, *J* = 8.4 Hz, 2H), 6.54 (dd, *J* = 18.0, 8.0 Hz, 2H), 5.63 (d, *J* = 10.2 Hz, 1H), 2.97 (s, 2H), 2.92 – 2.79 (m, 8H), 2.77 – 2.71 (m, 2H), 2.47 (t, *J* = 7.1 Hz, 2H), 2.22 – 2.01 (m, 4H), 1.70 (t, *J* = 7.4 Hz, 2H), 1.65 – 1.57 (m, 6H), 1.43 – 1.33 (m, 6H), 1.24 (d, *J* = 8.4 Hz, 54H), 1.12 (s, 2H), 0.86 (t, *J* = 6.7 Hz, 9H). HRMS (ESI, *m/z*): [M+H]^+^ calcd for C_64_H_110_N_3_O_7_S^+^,1064.7988; found, 1064.8063.

**SPAL3:** ^1^H NMR (400 MHz, Chloroform-*d*) δ 7.16 – 7.11 (m, 1H), 7.07 – 6.97 (m, 2H), 6.80 (dd, *J* = 8.7, 6.8 Hz, 2H), 6.58 – 6.49 (m, 2H), 6.41 (d, *J* = 2.3 Hz, 1H), 5.66 (d, *J* = 10.2 Hz, 1H), 3.67 (s, 2H), 3.10 (dd, *J* = 9.5, 4.5 Hz, 8H), 2.57 – 2.45 (m, 4H), 2.33 (q, *J* = 7.5 Hz, 2H), 2.03 (d, *J* = 7.6 Hz, 2H), 1.75 (s, 4H), 1.27 (d, *J* = 9.4 Hz, 68H), 0.88 (s, 9H), 0.86 (s, 3H). HRMS (ESI, *m/z*): [M]^+^ calcd for C_64_H_110_N_3_O_3_^+^,968.8542; found, 968.8553.

**SPAL4:** ^1^H NMR (500 MHz, Chloroform-d) δ 7.00 (d, J = 8.2 Hz, 1H), 6.78 (d, J = 10.3 Hz, 1H), 6.70 – 6.65 (m, 2H), 6.54 (s, 1H), 6.45 – 6.40 (m, 2H), 5.65 (d, J = 10.2 Hz, 1H), 3.78 (s, 3H), 3.09 (s, 2H), 3.00 – 2.92 (m, 8H), 2.88 (s, 4H), 2.52 (t, J = 7.2 Hz, 2H), 1.75 (s, 2H), 1.72 (s, 2H), 1.27 (d, J = 13.1 Hz, 65H), 1.15 (s, 3H), 0.88 (d, J = 4.2 Hz, 9H), 0.86 (s, 3H). HRMS (ESI, *m/z*): [M]^+^ calcd for C_65_H_112_N_3_O_4_^+^,998.8648; found, 998.8654.

**SPAL5:** ^1^H NMR (400 MHz, Chloroform-*d*) δ 7.03 (dd, *J* = 15.1, 7.7 Hz, 2H), 6.84 – 6.77 (m, 2H), 6.56 – 6.47 (m, 2H), 6.42 (d, *J* = 2.3 Hz, 1H), 3.19 – 3.03 (m, 4H), 2.80 – 2.65 (m, 8H), 2.58 – 2.48 (m, 6H), 1.96 – 1.81 (m, 4H), 1.72 (q, *J* = 7.7 Hz, 6H), 1.42 (s, 9H), 1.28 (s, 6H), 1.25 (s, 54H), 0.86 (d, *J* = 7.1 Hz, 9H). HRMS (ESI, *m/z*): [M]^+^ calcd for C_68_H_117_N_4_O_5_^+^,1069.9019; found, 1069.9028.

**SPAL6:** ^1^H NMR (400 MHz, Chloroform-*d*) δ 7.00 (d, *J* = 8.2 Hz, 1H), 6.79 (d, *J* = 10.3 Hz, 1H), 6.71 – 6.62 (m, 2H), 6.53 (d, *J* = 8.1 Hz, 1H), 6.42 (d, *J* = 8.4 Hz, 2H), 5.65 (d, *J* = 9.8 Hz, 1H), 3.76 (s, 3H), 2.95 (s, 4H), 2.82 (q, *J* = 9.0 Hz, 8H), 2.70 (s, 4H), 2.50 (t, *J* = 7.1 Hz, 2H), 2.11 (s, 2H), 1.74 – 1.66 (m, 6H), 1.41 (s, 9H), 1.28 (s, 6H), 1.24 (s, 54H), 0.86 (d, *J* = 7.1 Hz, 9H). HRMS (ESI, *m/z*): [M]^+^ calcd for C_69_H_119_N_4_O_6_^+^,1099.9124; found, 1099.9194.

Synthesis of **compound SP**

A solution of **compound 7a** (100 mg, 0.36 mmol) and Salicylaldehyde (43.4 mg, 0.36 mmol) was added to 5 mL EtOH. The reaction was stirred at 80 °C for 6 hours. Upon completion, the organic phase was concentrated under reduced pressure. The yellow solid is **compound SP1** (87 mg, 64%). **Compound SP2-SP6** was synthesized using a similar method to that of **compound SP1**.

**SP1**: ^1^H NMR (500 MHz, DMSO-*d*_6_) δ 11.03 (s, 1H), 8.60 (d, *J* = 16.3 Hz, 1H), 8.28 (dd, *J* = 8.0, 1.7 Hz, 1H), 8.05 – 8.00 (m, 1H), 7.92 – 7.83 (m, 2H), 7.67 – 7.57 (m, 2H), 7.48 (ddd, *J* = 8.4, 7.1, 1.7 Hz, 1H), 7.04 (d, *J* = 8.2 Hz, 1H), 6.99 (t, *J* = 7.6 Hz, 1H), 4.81 (t, *J* = 7.9 Hz, 2H), 2.65 (t, *J* = 6.5 Hz, 2H), 2.18 (h, *J* = 6.2, 5.6 Hz, 2H), 1.77 (s, 6H). HRMS (ESI, *m/z*): [M+H]^+^ calcd for C_21_H_24_NO_4_S^+^,386.1348; found, 386.1423.

**SP2**:^1^H NMR (400 MHz, DMSO-*d*_6_) δ 10.92 (s, 1H), 8.50 (d, *J* = 16.4 Hz, 1H), 8.24 (dd, *J* = 8.1, 1.6 Hz, 1H), 7.95 (dd, *J* = 8.9, 1.9 Hz, 1H), 7.81 (d, *J* = 16.4 Hz, 1H), 7.51 (d, *J* = 2.5 Hz, 1H), 7.46 – 7.42 (m, 1H), 7.18 – 7.14 (m, 1H), 7.02 (dd, *J* = 8.4, 1.1 Hz, 1H), 6.96 (d, *J* = 7.6 Hz, 1H), 6.63 (t, *J* = 9.2 Hz, 1H), 4.77 (t, *J* = 7.8 Hz, 2H), 3.89 (s, 3H), 2.62 (q, *J* = 6.1 Hz, 2H), 2.15 (s, 2H), 1.76 (s, 6H). HRMS (ESI, *m/z*): [M+H]^+^ calcd for C_22_H_26_NO_5_S^+^,416.1453; found, 416.1523.

**SP3**: ^1^H NMR (400 MHz, DMSO-*d*_6_) δ 11.16 (s, 1H), 8.55 (d, *J* = 16.2 Hz, 1H), 8.12 (d, *J* = 7.9 Hz, 1H), 7.98 – 7.92 (m, 1H), 7.92 – 7.85 (m, 1H), 7.79 (d, *J* = 16.3 Hz, 1H), 7.66 – 7.58 (m, 2H), 7.49 (t, *J* = 7.9 Hz, 1H), 7.03 (dd, *J* = 13.0, 5.4 Hz, 2H), 4.60 (t, *J* = 7.6 Hz, 2H), 1.85 (t, *J* = 7.6 Hz, 2H), 1.78 (s, 6H), 1.45 (t, *J* = 7.8 Hz, 2H), 0.95 (t, *J* = 7.3 Hz, 3H). HRMS (ESI, *m/z*): [M]^+^ calcd for C_22_H_26_NO^+^,320.2009; found, 320.2012.

**SP4**:^1^H NMR (400 MHz, DMSO-*d*_6_) δ 7.15 (d, *J* = 7.6 Hz, 1H), 7.09 – 7.05 (m, 2H), 6.96 (d, *J* = 10.1 Hz, 1H), 6.82 (t, *J* = 7.4 Hz, 2H), 6.74 (t, *J* = 7.4 Hz, 1H), 6.63 (d, *J* = 8.1 Hz, 1H), 6.54 (d, *J* = 7.9 Hz, 1H), 5.75 (s, 1H), 3.17 (q, *J* = 7.6 Hz, 2H), 2.94 (dt, *J* = 13.0, 6.9 Hz, 3H), 1.66 (dd, *J* = 17.3, 9.1 Hz, 2H), 1.37 (s, 6H), 1.26 (s, 3H), 1.19 (s, 2H). HRMS (ESI, *m/z*): [M]^+^ calcd for C_23_H_28_NO_2_^+^,350.2115; found, 350.2118.

**SP5**: ^1^H NMR (500 MHz, DMSO-*d*_6_) δ 8.57 (s, 1H), 8.18 (s, 1H), 7.88 (s, 2H), 7.54 (d, *J* = 68.1 Hz, 3H), 7.04 (d, *J* = 44.4 Hz, 3H), 4.64 (s, 2H), 4.12 (s, 2H), 3.44 (s, 2H), 1.77 (s, 6H), 1.29 (t, *J* = 15.6 Hz, 9H). HRMS (ESI, *m/z*): [M]^+^ calcd for C_26_H_33_N_2_O_3_^+^,421.2486; found, 421.2484.

**SP6**:^1^H NMR (400 MHz, DMSO-*d*_6_) δ 10.98 (s, 1H), 8.49 (d, *J* = 16.3 Hz, 1H), 8.14 – 8.08 (m, 1H), 7.82 (d, *J* = 8.9 Hz, 1H), 7.63 (d, *J* = 16.4 Hz, 1H), 7.54 (d, *J* = 2.4 Hz, 1H), 7.51 – 7.42 (m, 1H), 7.16 (dd, *J* = 8.9, 2.4 Hz, 1H), 7.06 (d, *J* = 8.3 Hz, 1H), 6.98 (d, *J* = 7.6 Hz, 1H), 4.60 (t, *J* = 7.3 Hz, 2H), 3.89 (s, 3H), 3.09 (q, *J* = 6.3 Hz, 2H), 2.01 (p, *J* = 6.9 Hz, 2H), 1.77 (s, 6H), 1.32 (s, 9H). HRMS (ESI, *m/z*): [M]^+^ calcd for C_27_H_35_N_2_O_4_^+^,451.2591; found, 4251.2590.

1. **Light-Triggered Proton Release of Spiropyran**

Spiropyran was dissolved in a water/ethanol (70:30, v/v) mixture, and the pH was adjusted to a weakly acidic condition. The pH values were recorded before and after 5 min irradiation with a 460 nm xenon lamp. In both cases, a decrease in pH was observed, accompanied by a color change from yellow to pale yellow. These results indicate that upon photoirradiation, spiropyran undergoes an open-ring-to-closed-ring isomerization, accompanied by the release of a proton.


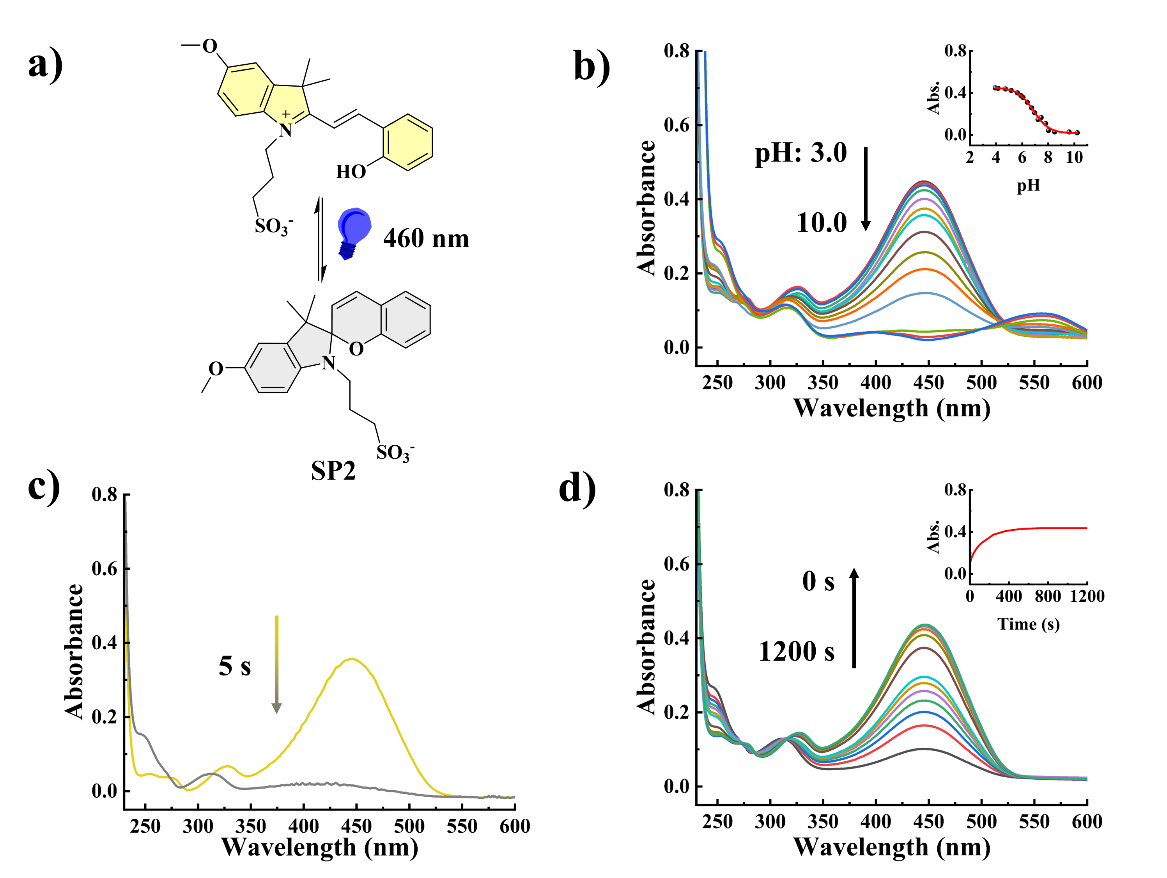


**Figure S1.** Absorption spectrum of SP2. a) SP2 ring-opening and ring-closing schematic diagram. b) Absorption spectra under different pH conditions (yellow, λ_MCH_ = 430-460 nm; purple, λ_MC_ = 500-600 nm). Experimental conditions: [SP] = 30 mM, pH: 3-10, T = 25 °C, c) Absorption spectra before and after illumination, *hv* (blue, 460 nm, 5 W, 10 s), d) The changes of absorption spectra after illumination, time:1200 s.


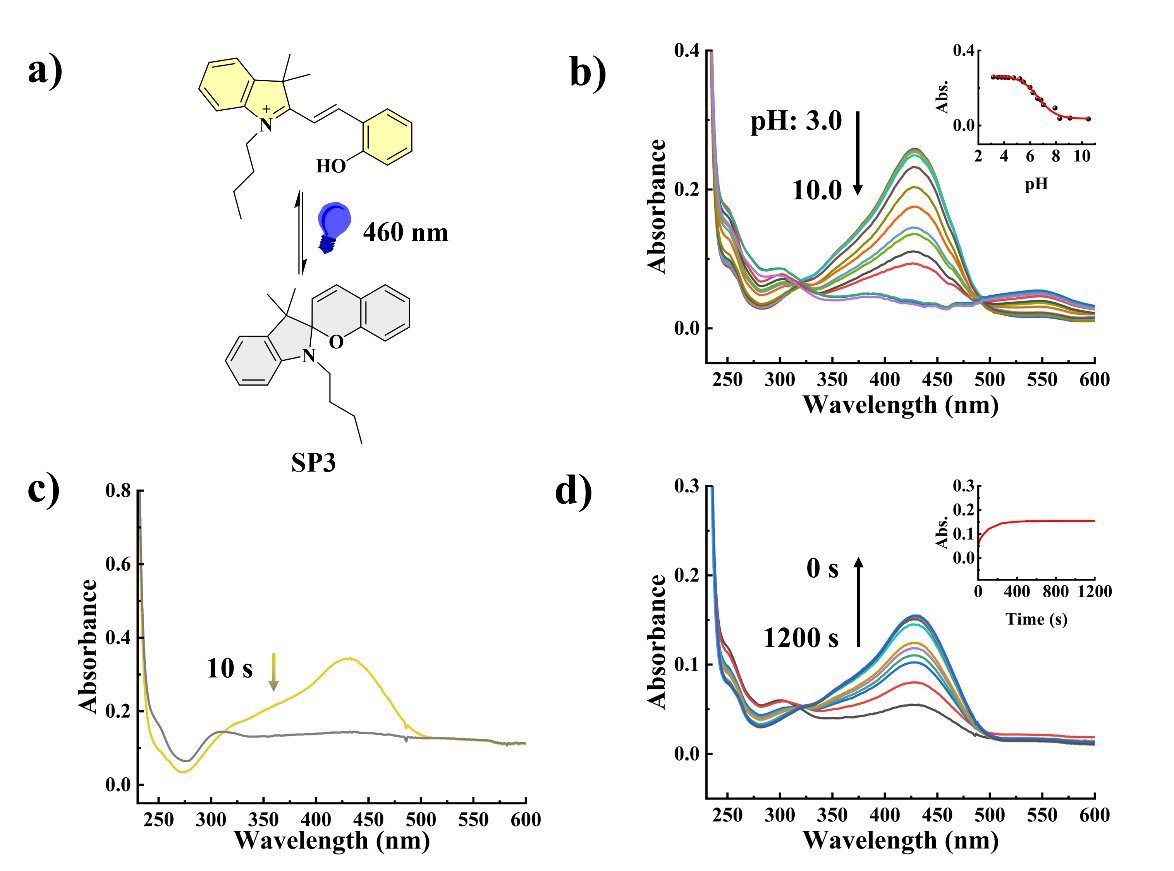


**Figure S2.** Absorption spectrum of SP3. a) SP3 ring-opening and ring-closing schematic diagram. b) Absorption spectra under different pH conditions (yellow, λ_MCH_ = 430-460 nm; purple, λ_MC_ = 500-600 nm). Experimental conditions: [SP] = 30 mM, pH: 3-10, T = 25 °C, c) Absorption spectra before and after illumination, *hv* (blue, 460 nm, 5 W, 10 s), d) The changes of absorption spectra after illumination, time: 1200 s.


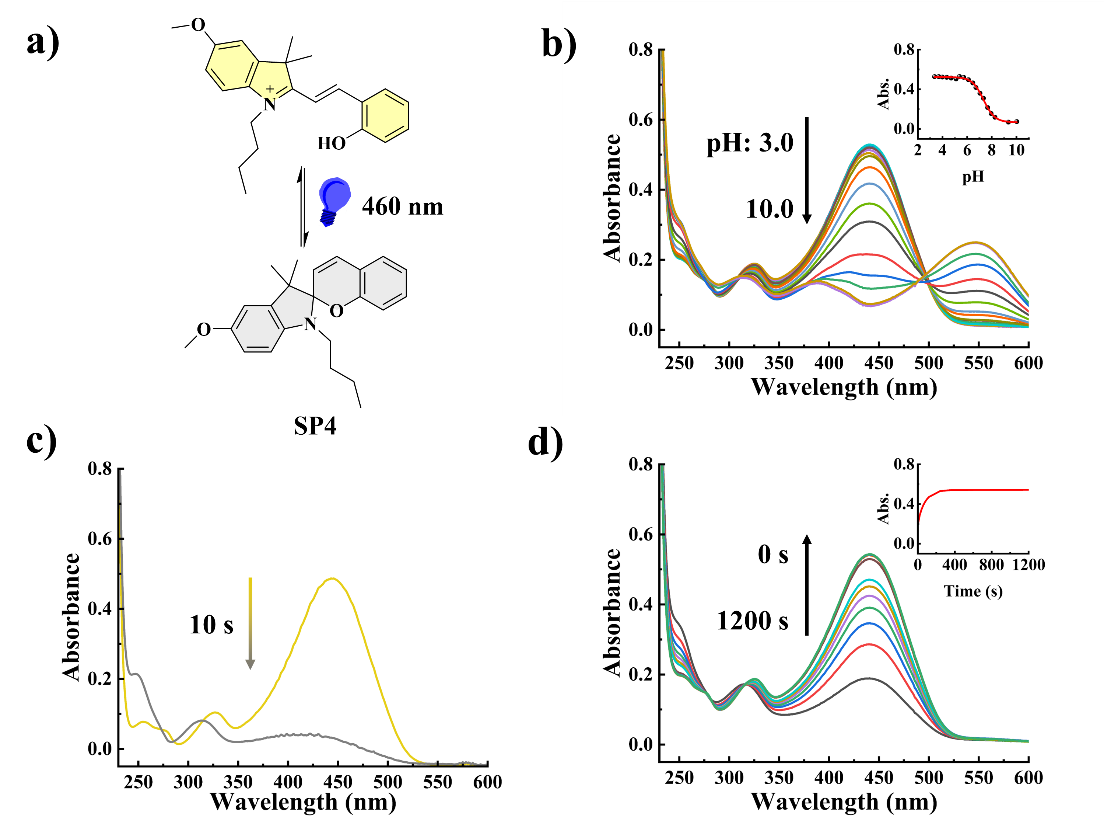


**Figure S3.** Absorption spectrum of SP4. SP4 ring-opening and ring-closing schematic diagram. b) Absorption spectra under different pH conditions (yellow, λ_MCH_ = 430-460nm; purple, λ_MC_ = 500-600 nm). Experimental conditions: [SP] = 30 mM, pH: 3-10, T = 25 °C, c) Absorption spectra before and after illumination, *hv* (blue, 460 nm, 5 W, 10 s), d) The changes of absorption spectra after illumination, time: 1200 s.


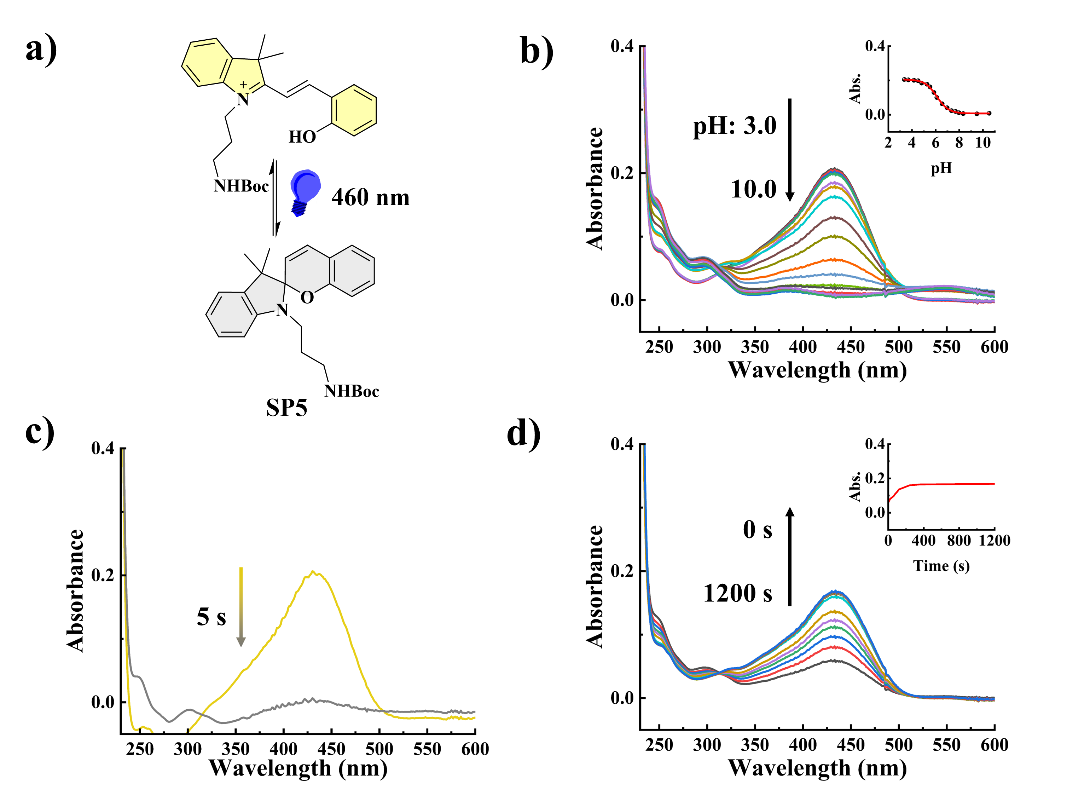


**Figure S4.** Absorption spectrum of SP5. a) SP5 ring-opening and ring-closing schematic diagram. b) Absorption spectra under different pH conditions (yellow, λ_MCH_ = 430-460 nm; purple, λ_MC_ = 500-600 nm). Experimental conditions: [SP] = 30 mM, pH: 3-10, T = 25 °C, c) Absorption spectra before and after illumination, *hv* (blue, 460 nm, 5 W, 10 s), d) The changes of absorption spectra after illumination, time: 1200 s.


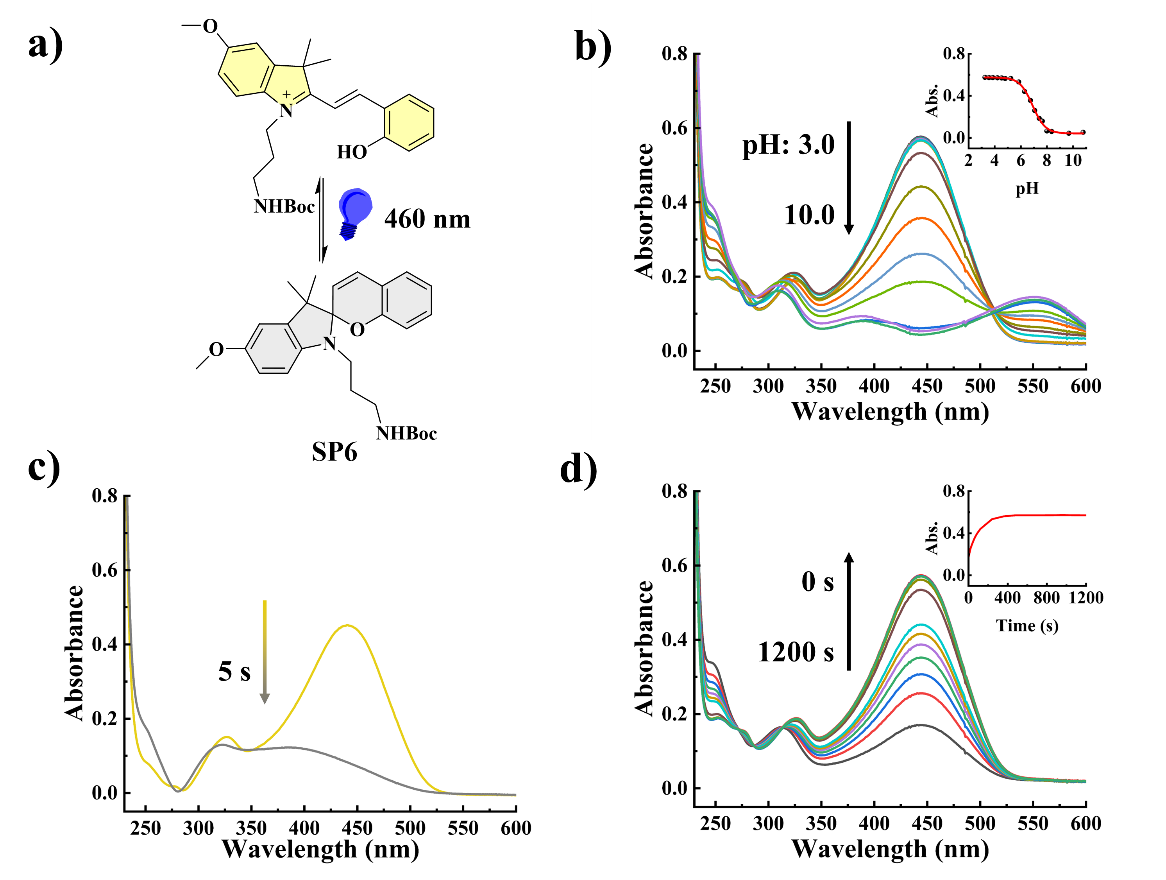


**Figure S5.** Absorption spectrum of SP6. a) SP6 ring-opening and ring-closing schematic diagram. b) Absorption spectra under different pH conditions (yellow, λ_MCH_ = 430-460 nm; purple, λ_MC_ = 500-600 nm). Experimental conditions: [SP] = 30 mM, pH: 3-10, T = 25 °C, c) Absorption spectra before and after illumination, *hv* (blue, 460 nm, 5 W, 10 s), d) The changes of absorption spectra after illumination, time: 1200 s.

1. **Preparation of Nanoparticles**

All chemicals were purchased from Energy Chemical Co., Ltd. (Shanghai, China) and used without further purification. DOPE (1,2-dioleoyl-sn-glycero-3-phosphoethanolamine) and cholesterol were supplied by Aladdin Biochemical Technology Co., Ltd. (Shanghai, China). DMG-PEG 2000 was purchased from Avanti Polar Lipids, Inc. (Alabaster, AL, USA). Nanoparticles were prepared using the pipette-based mixing method. For nanoparticle characterization, including particle size analysis, nanoparticles encapsulating ctDNA were used. For transfection experiments, nanoparticles were loaded with mRNA fragments encoding green fluorescent protein (eGFP). Both types of nanoparticles were prepared using the same protocol.


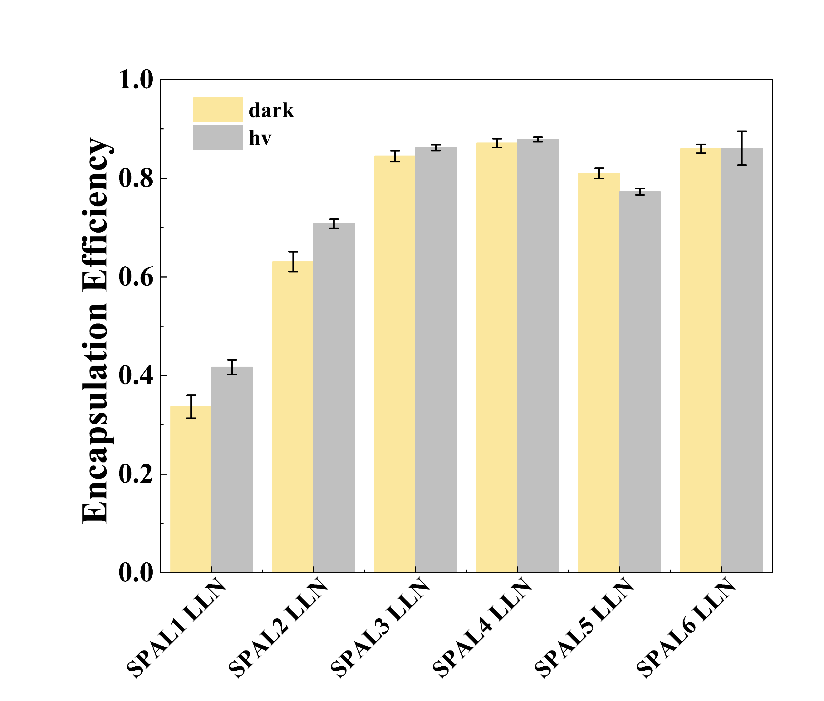


**Figure S6.** Encapsulation Efficiency of Spiropyran Nanoparticles

1. **Cell Culture**

Raw 264.7 (Macrophage cells) are obtained from the Institute of Basic Medical Sciences (IBMS) of the Chinese Academy of Medical Sciences (CAMS). All cell lines are maintained under standard culture conditions (an atmosphere of 5% CO_2_ and 95% air at 37 °C) in DMEM medium supplemented with 10% fetal bovine serum (FBS).

MTT assay

RAW 264.7 (Macrophage cells) were cultured in DMEM supplemented with 10% FBS (fetal bovine serum) in an atmosphere of 5% CO_2_ and 95% air at 37 °C. For mRNA MTT assays, macrophage cells in the exponential growth phase were plated into 96-wells board containing 100 μl DMEM per compartment. The media was removed after incubation at 37 °C with 5% CO_2_ for 24 hours. A freshly prepared nanoparticle stock solution was then added to the DMEM culture medium. After incubation for 3.5 hours, the cells were exposed to 460 nm LED light for 5 minutes, followed by an additional 21-hour incubation. After adding MTT reagent, let it stand for 4 hours, then dissolve the purple crystals in DMSO, and detect the absorbance at 570 nm using microplate reader.


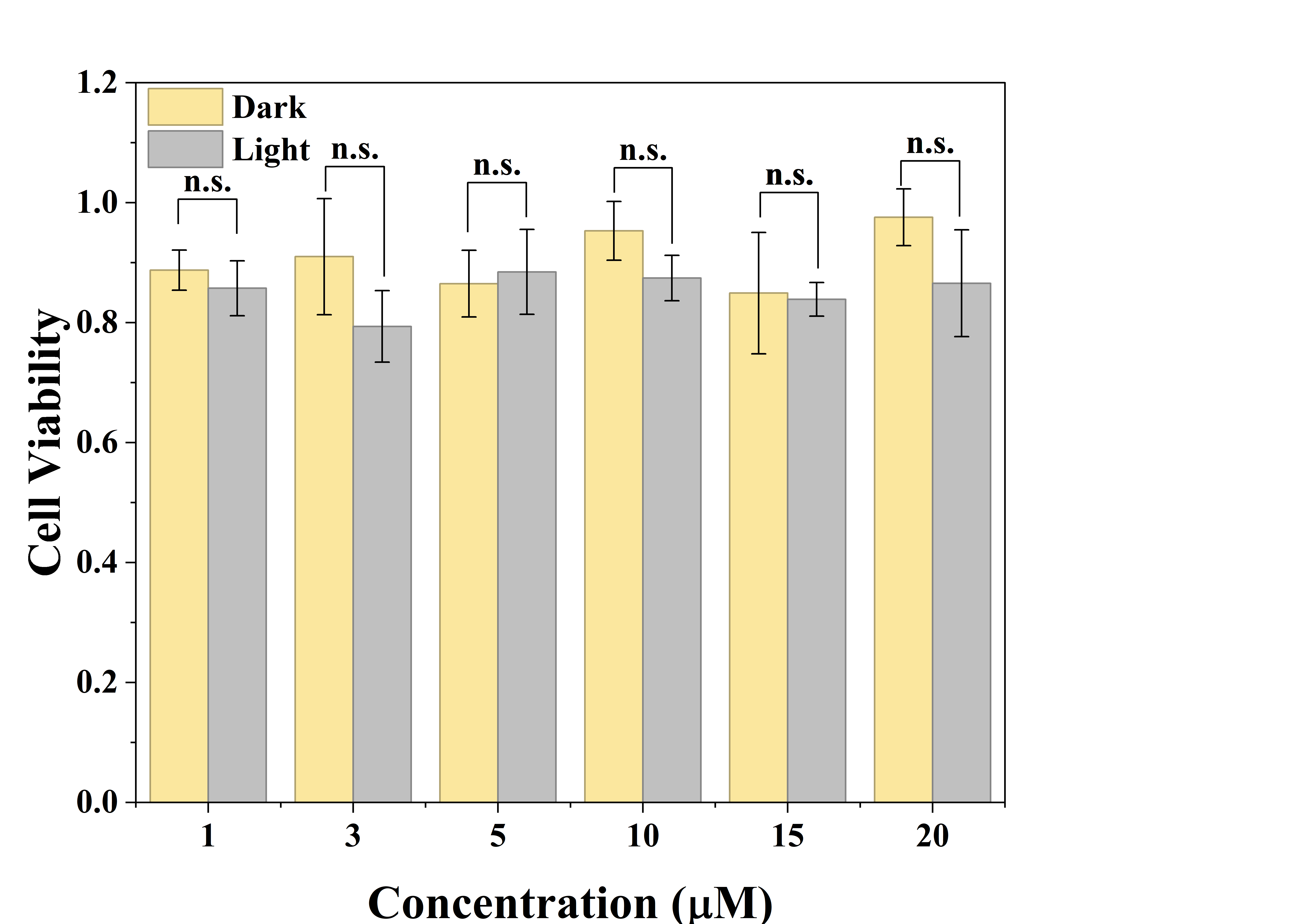


**Figure S7.** MTT assay of cells with various concentrations of SPAL1,with and without illumination.

Transfection method for Spiropyran-Lipid

RAW 264.7 (Macrophage cells) were cultured in DMEM supplemented with 10% FBS (fetal bovine serum) in an atmosphere of 5% CO_2_ and 95% air at 37 °C. For mRNA transfection studies, macrophage cells in the exponential growth phase were plated into a 35 mm cross-shaped compartmentalized confocal dish containing 500 μl DMEM per compartment. The media was removed after incubation at 37 °C with 5% CO_2_ for 24 hours. A freshly prepared nanoparticle stock solution was then added to the DMEM culture medium. After incubation for 3.5 hours, the cells were exposed to 460 nm LED light for 5 minutes, followed by an additional 6-hour incubation. Confocal imaging was then performed using a confocal laser scanning microscope.

In terms of cell culture procedures, the experimental operations for flow cytometry were essentially the same as those for confocal microscopy imaging. The culture medium was removed after washing with 500 mL of PBS, and the cells were resuspended in 300 μL of culture medium in a centrifuge tube. Samples were illuminated with a sapphire laser at 488 nm on a FACScan flow cytometer. Each group of samples detected a total of 10000 cells in 1 min. Analysis of flow cytometry data with FlowJo software.


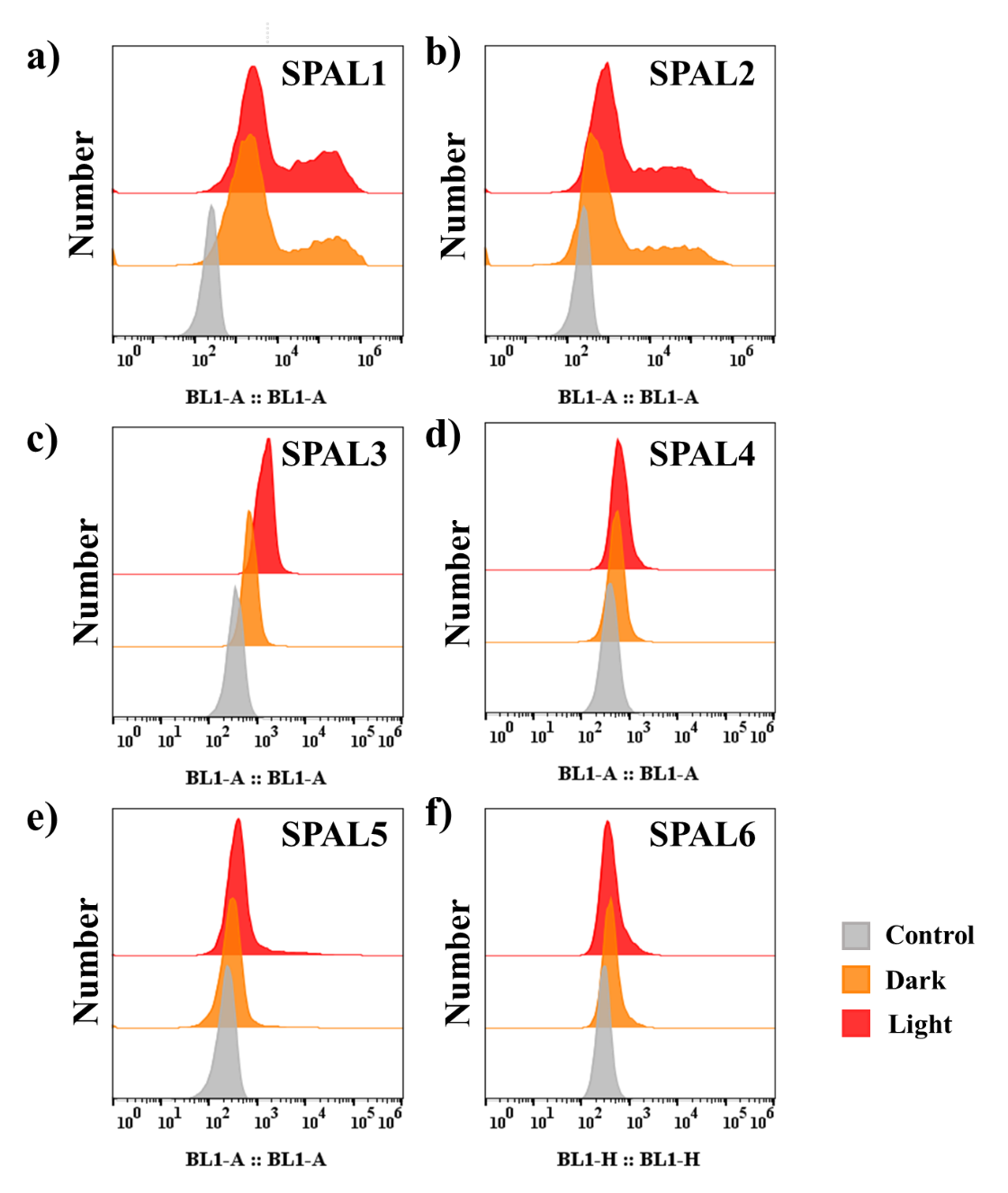


**Figure S8.** Flow cytometry analysis showing green fluorescence-positive cells (grey: control, orange: dark, red: light)

**Table S1.** Transfection Efficiency of SPALs

|  | eGFP Positive Rate (%) | | | eGFP Average Intensity | |
| --- | --- | --- | --- | --- | --- |
|  | Control | Dark | Light | Dark | Light |
| SPAL1 | 0.71 | 59.37 | 72.1 | 96813.00 | 82873.00 |
| SPAL2 | 0.75 | 44.4 | 64.5 | 43000.67 | 25533.67 |
| SPAL3 | 0.07 | 0.53 | 3.15 | 903.00 | 1628.67 |
| SPAL4 | 0.69 | 0.09 | 0.18 | 2241.00 | 1695.67 |
| SPAL5 | 0.62 | 2.37 | 6.14 | 35135.67 | 30242.67 |
| SPAL6 | 0.38 | 0.2 | 0.6 | 38927.00 | 72777.33 |

RAW 264.7 (Macrophage cells) were cultured in DMEM supplemented with 10% FBS (fetal bovine serum) in an atmosphere of 5% CO_2_ and 95% air at 37 °C. For mRNA transfection studies, macrophage cells in the exponential growth phase were plated into a 35 mm cross-shaped compartmentalized confocal dish containing 500 μl DMEM per compartment. The media was removed after incubation at 37 °C with 5% CO_2_ for 24 hours. Under identical nucleic acid dosing conditions, transfection mediated by Lipofectamine™ 3000 (Thermo Fisher Scientific) showed no apparent change in efficiency upon light irradiation, suggesting that light exposure alone does not affect transfection efficiency **(Figure S9)**.


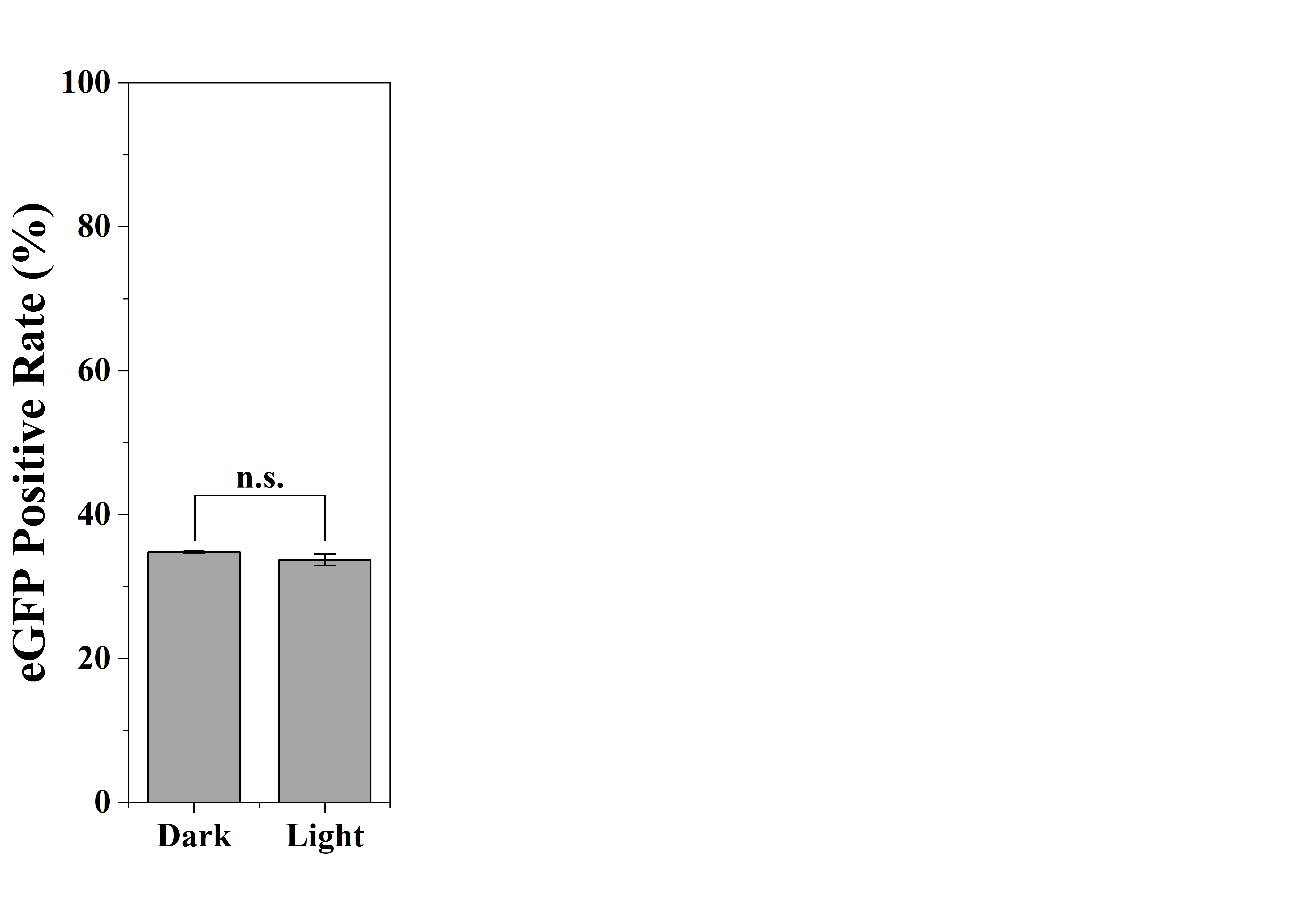


**Figure S9.** eGFP positive of lipo3000 with and without illumination

**Co-transfection method for Spiropyran-Lipid and Ratiometric Fluorescent Probe-Lipid Materials**

During the preparation of nanoparticles, a fluorescent probe-lipid conjugate was incorporated into the ethanol phase at a ratio of 4% (w/w). The remaining steps followed the standard nanoparticle formulation protocol. RAW 264.7 (Macrophage cells) were cultured in DMEM supplemented with 10% FBS (fetal bovine serum) in an atmosphere of 5% CO_2_ and 95% air at 37 °C. For co-transfection studies, macrophage cells in the exponential growth phase were plated into a 35 mm cross-shaped compartmentalized confocal dish containing 500 μl DMEM per compartment. The media was removed after incubation at 37 °C with 5% CO_2_ for 24 hours. A freshly prepared nanoparticle stock solution was then added to the DMEM culture medium. After incubation for 3.5 hours, confocal imaging was performed using a Zeiss confocal microscope with an excitation wavelength of 488  nm. The samples were then exposed to 460 nm LED light for 5 minutes, followed immediately by a second round of confocal imaging under the same conditions.


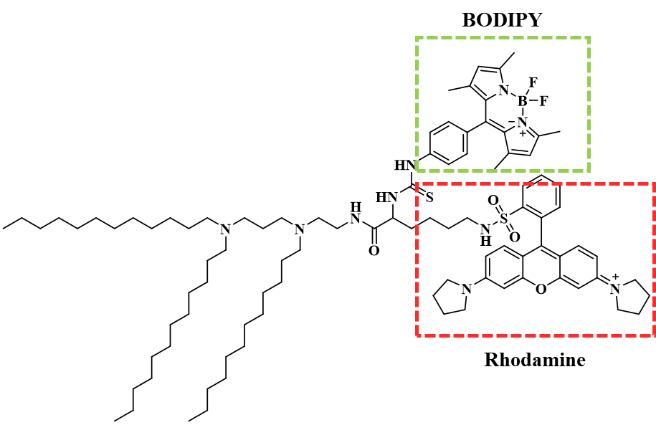


**Figure S10.** Schematic illustration of the ratiometric fluorescent probe structure.

**Table S2.** Mean Endosomal Fluorescence Intensity before and after illumination.

| Dark | | | Light | | | Rate of Ratio Increase |
| --- | --- | --- | --- | --- | --- | --- |
| Green channel | Red channel | Ratio | Green channal | Red channel | Ratio |  |
| 36.17 | 55.53 | 1.54 | 24.63 | 45.92 | 1.86 | 0.21 |
| 84.70 | 122.87 | 1.45 | 69.53 | 111.38 | 1.60 | 0.10 |
| 26.63 | 23.83 | 0.89 | 48.74 | 52.73 | 1.08 | 0.21 |
| 7.26 | 15.71 | 2.16 | 25.02 | 55.69 | 2.23 | 0.03 |
| 31.18 | 33.61 | 1.08 | 34.37 | 53.29 | 1.55 | 0.44 |
| 31.69 | 34.90 | 1.10 | 47.24 | 55.41 | 1.17 | 0.07 |
| 26.86 | 34.04 | 1.27 | 30.61 | 54.14 | 1.77 | 0.40 |
| 23.13 | 25.83 | 1.12 | 32.33 | 66.55 | 2.06 | 0.84 |
| 15.05 | 29.18 | 1.94 | 41.97 | 84.93 | 2.02 | 0.04 |

1. **^1^H, ^13^C NMR Spectra of SPAL**


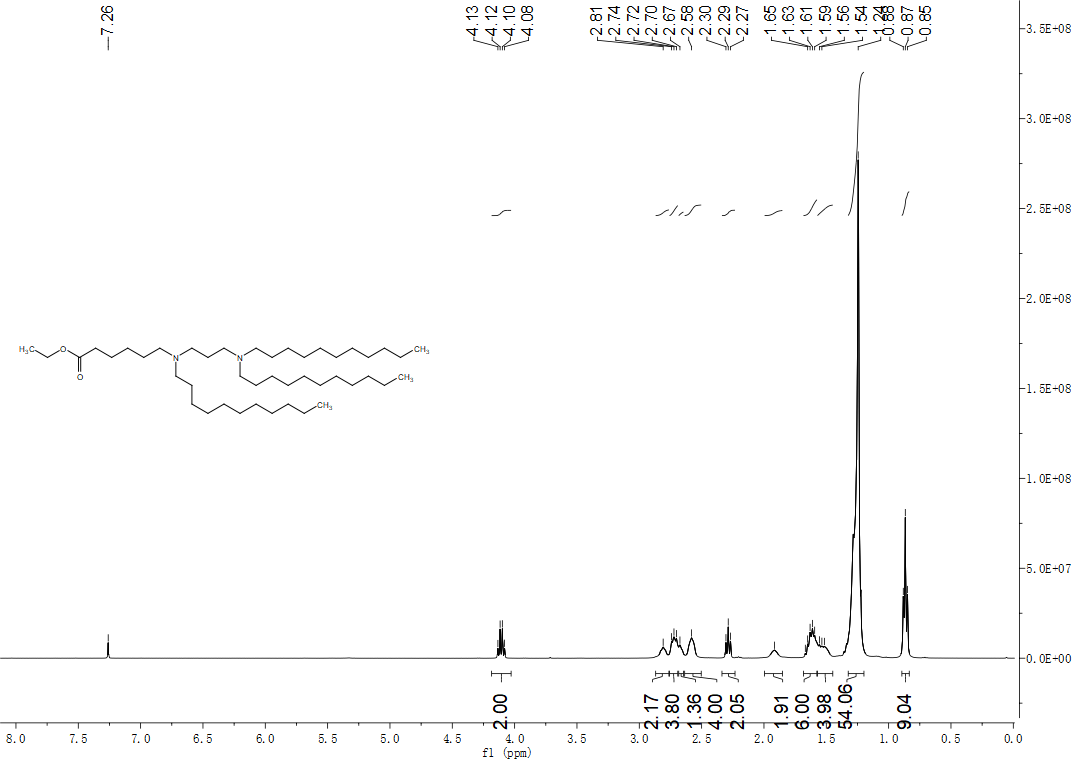


**Figure S11.** ^1^H NMR Spectra of **compound 4**

**Figure S12.** ^1^H NMR Spectra of **compound 8a**

**Figure S13.** High resolution mass spectrum of **compound 8a**

**Figure S14.** ^1^H NMR Spectra of **compound SPAL1**

**Figure S15.** High resolution mass spectrum **compound SPAL1**

**Figure S16.** ^1^H NMR Spectra of **compound SPAL2**

**Figure S17.** High resolution mass spectrum **compound SPAL2**

**Figure S18.** ^1^H NMR Spectra of **compound SPAL3**

**Figure S19.** High resolution mass spectrum **compound SPAL3**

**Figure S20.** ^1^H NMR Spectra of **compound SPAL4**

**Figure S21.** High resolution mass spectrum **compound SPAL4**

**Figure S22.** ^1^H NMR Spectra of **compound SPAL5**

**Figure S23.** High resolution mass spectrum **compound SPAL5**

**Figure S24.** ^1^H NMR Spectra of **compound SPAL6**

**Figure S25.** High resolution mass spectrum **compound SPAL6**

**Figure S26.** ^1^H NMR Spectra of **compound SP1**

**Figure S27.** High resolution mass spectrum **compound SP1**

**Figure S28.** ^1^H NMR Spectra of **compound SP2**

**Figure S29.** High resolution mass spectrum **compound SP2**

**Figure S30.** ^1^H NMR Spectra of **compound SP3**

**Figure S31.** High resolution mass spectrum **compound SP3**

**Figure S32.** ^1^H NMR Spectra of **compound SP4**

**Figure S33.** High resolution mass spectrum **compound SP4**

**Figure S34.** ^1^H NMR Spectra of **compound SP5**

**Figure S35.** High resolution mass spectrum **compound SP5**

**Figure S36.** ^1^H NMR Spectra of **compound SP6**

**Figure S37.** High resolution mass spectrum **compound SP6**
